# Methylations in dilated cardiomyopathy and heart failure

**DOI:** 10.3389/fcvm.2025.1559550

**Published:** 2025-04-11

**Authors:** Cong Qin, Yansong Qin, Shanshan Zhou

**Affiliations:** ^1^ Department of Cardiology, The First Hospital of Jilin University, Changchun, China; ^2^ Undergraduate School, Shanghai Jiao Tong University School of Medicine, Shanghai, China

**Keywords:** dilated cardiomyopathy, methylation, cardiac hypertrophy, heart failure, m6A

## Abstract

Dilated cardiomyopathy (DCM) is characterized by impaired expansion or contraction of the left or both ventricles in the absence of abnormal load conditions (such as primary valve disease) or severe coronary artery disease that can lead to ventricular remodeling. Genetic mutations, infections, inflammation, autoimmune diseases, exposure to toxins, and endocrine or neuromuscular factors have all been implicated in the causation of DCM. Cardiomyopathy, particularly DCM, often has genetic underpinnings, with established or suspected genetic origins. Up to 40% of DCM cases involve probable or confirmed genetic variations. The significance of RNA modification in the pathogenesis of hypertension, cardiac hypertrophy, and atherosclerosis is well-established. Of late, RNA methylation has garnered attention for its involvement in DCM. This review examines the biological mechanisms and effects of RNA methylation in DCM and heart failure.

## Introduction

Dilated cardiomyopathy (DCM) is characterized by enlargement of one or both ventricles, accompanied by dysfunction of the myocardial contractile apparatus. The condition often results in heart failure. DCM can be further subdivided into primary DCM and secondary DCM. Several factors have been implicated in the etiopathogenesis of DCM. Genetic predisposition is a unique feature of this condition. Approximately 25%–50% of patients are diagnosed with primary DCM (1). Heymans et al*.* reported heritable genetic mutations in 5%–15% of patients with secondary DCM (2). Thus, implementing strategies for the prevention, screening, and diagnosis of familial DCM is a key imperative.

In addition to genetic factors, the etiology of dilated cardiomyopathy encompasses acute myocarditis, viral or bacterial infections, toxin exposure, and the natural aging process. Furthermore, persistent chronic myocarditis may precipitate the development of secondary DCM (3). Pauschinger et al. conducted PCR amplification of the DCM genome and found a large number of samples testing positive for enterovirus and EBV (4). They concluded that EBV can cause lymphadenopathy and splenomegaly and can lead to peripheral blood monocytosis or heterogeneous lymphocytes. Nevertheless, few studies have demonstrated an abnormal increase in monocytes in the myocardium, or an abnormal increase in macrophage density.

The hallmark of DCM is systolic dysfunction, predominantly due to cardiac dilatation and a reduced left ventricular ejection fraction (5). The initial stage of DCM is marked by left ventricular dilatation, which subsequently leads to enlargement of the chambers. This impairs the contractility of the right ventricle and atria. The reduced pumping capacity triggers compensatory mechanisms giving rise to arrhythmias and progressive heart failure (6). Furthermore, chamber dilatation and slowed blood flow may precipitate thrombosis, thereby exacerbating the pumping impairment (7, 8).

Patients with end-stage DCM exhibit stagnant venous systems and inadequate arterial perfusion (9). Heart failure in patients with DCM is associated with mitochondrial dysfunction (10). Furthermore, abnormal decrease in choline kinase also affects cardiac metabolism in patients with DCM/heart failure (7, 11). Additionally, cardiac fibrosis and cytoskeletal disruption are also involved in the pathophysiology (12, 13). A comprehensive analysis of the etiology, progression, and treatment of DCM cannot be achieved without considering heart failure, as the ultimate goal of treating DCM is to halt myocardial damage, control heart failure and arrhythmias, prevent sudden death and embolism and improve the quality of life and survival. The prognosis of patients with DCM is closely related to the severity of heart failure. From a molecular perspective, investigating the common molecular mechanisms underlying DCM and heart failure can facilitate the early identification of these mechanisms, enabling timely interventions to stabilize the condition and impede its progression.

**Figure 1:**
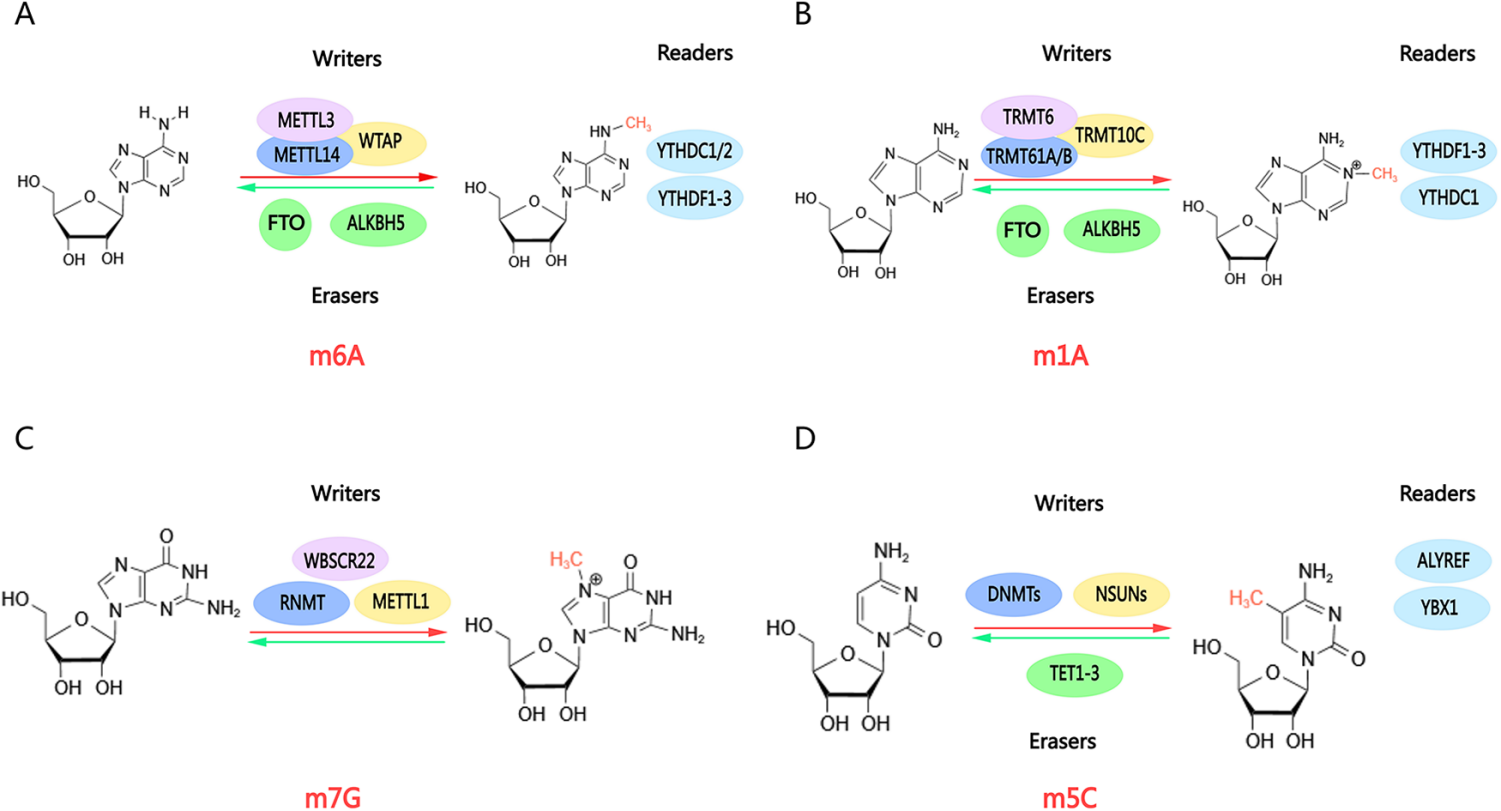
Molecular structures of RNA methylations. RNA methylations are dynamic modifications mediated by enzymes, including methylases (“writers”), demethylases (“erasers”), and methylation recognition enzymes (“readers”). Methylases and demethylases coordinate reversible changes in RNA methylation, enabling methylation recognition enzymes to read methylation sites and execute subsequent functions. **(A)** m6A; **(B)** m1A; **(C)** m7G; **(D)** m5C. Created with MedPeer (medpeer.cn).

RNA methylation is an epigenetic modification mechanism whereby methyl groups are added to RNA strands. Methylation plays a significant role in regulating several key processes, including gene expression, RNA stability, splicing, transport, and translation (9). The most common RNA methylations in cardiovascular diseases are m1A, m6A, and m7G (Figure 1). m6A is the most prevalent form of m6A and has been shown to influence cardiomyocyte remodeling, hasten the progression of heart failure, stimulate autophagy, and inhibit apoptosis (14–16). Consequently, RNA methylation in cardiovascular disease can impact the efficiency of transcription and translation, alter the level of reactants, enhance or inhibit the response pathway, and ultimately manifest as fibrosis, hypertrophy, and dilatation.

The recent upsurge in RNA methylation research has shed light on its potential involvement in cardiovascular disease pathogenesis. However, a notable knowledge gap exists regarding its role in DCM, whereas studies abound on other conditions, such as hypertension, pulmonary hypertension, and vascular calcification. DCM, a complex and multifactorial disease with rising prevalence, presents a unique opportunity for exploration. Emerging evidence suggests a molecular link between RNA methylation and DCM, warranting further research. As the initial symptom of DCM is an enlargement of the left ventricular cavity, the increased compensatory effect results in a degree of enlargement of the cardiomyocytes, comparable to that observed in cardiac hypertrophy. The subsequent symptom of advanced DCM is heart failure. The present study focused on the role and mechanism of RNA methylation modifications (m6A, m5C, and m1A) in DCM, aiming to uncover shared molecular effects and signaling pathways underlying cardiac hypertrophy, DCM, and heart failure. This will facilitate the identification of molecular characteristics, biological functions, and impacts at the pathological level.

## Various methylations in cardiac hypertrophy

### M6A methylations in cardiac hypertrophy

N6-methyladenosine (m6A) RNA methylation is mediated by three distinct complexes: writers, erasers, and readers (Figure 1A). The writers include METTL3, METTL14, and WTAP (17). METTL3 serves as the catalytic subunit, regulating RNA methylation through its enzymatic activity. METTL14 enhances the catalytic properties of METTL3, While WTAP binds to METTL3 and METTL14 to amplify m6A methylation levels (18). Writers regulate various cellular processes including cellular reprogramming, reproductive function, immune function, and the endothelial‒hematopoietic transition (19–21). The erasers, comprising FTO and ALKBH5 demethylases, are responsible for removing methyl groups from RNA fragments. FTO has been implicated in the development of cardiac hypertrophy. Modulating FTO expression may offer a potential therapeutic strategy for managing early heart failure (22). ALKBH5 plays a pivotal role in localizing gene modifications, influencing mRNA synthesis and splicing (17, 23). Subsequently, YTHDF family members YTHDC1 and YTHDC2 act as readers, mediating reversible dynamic modifications. These reader proteins regulate RNA stability and translation, governing the metabolism of m6A-methylated mRNAs. YTHDF1 facilitates mRNA decay, whereas YTHDF2 transports targeted mRNAs to the cytoplasm for processing and degradation. This intricate process is important in DCM and heart failure (24, 25).

Cardiac hypertrophy, a prevalent pathological phenomenon in cardiovascular disease patients, is characterized by increased cardiomyocyte size in response to stress overload or other stimuli, aiming to enhance cardiac function. However, pathological cardiac hypertrophy leads to detrimental morphological and metabolic alterations, including cardiomyocyte fibrosis and mitochondrial dysfunction (26). Additionally, it may result in macroscopic structural modifications in myocardial segments and impaired angiogenesis. Furthermore, diabetes mellitus has been shown to induce cardiac hypertrophy (27) through mechanisms involving insulin resistance, disrupted fatty acid metabolism, and dyshomeostasis. The regulation of m6A methylation plays a crucial role in cardiac hypertrophy, modulating the expression and degradation of key genes and noncoding RNAs, including the METTL family and the ALKBH5 family.

**Figure 2:**
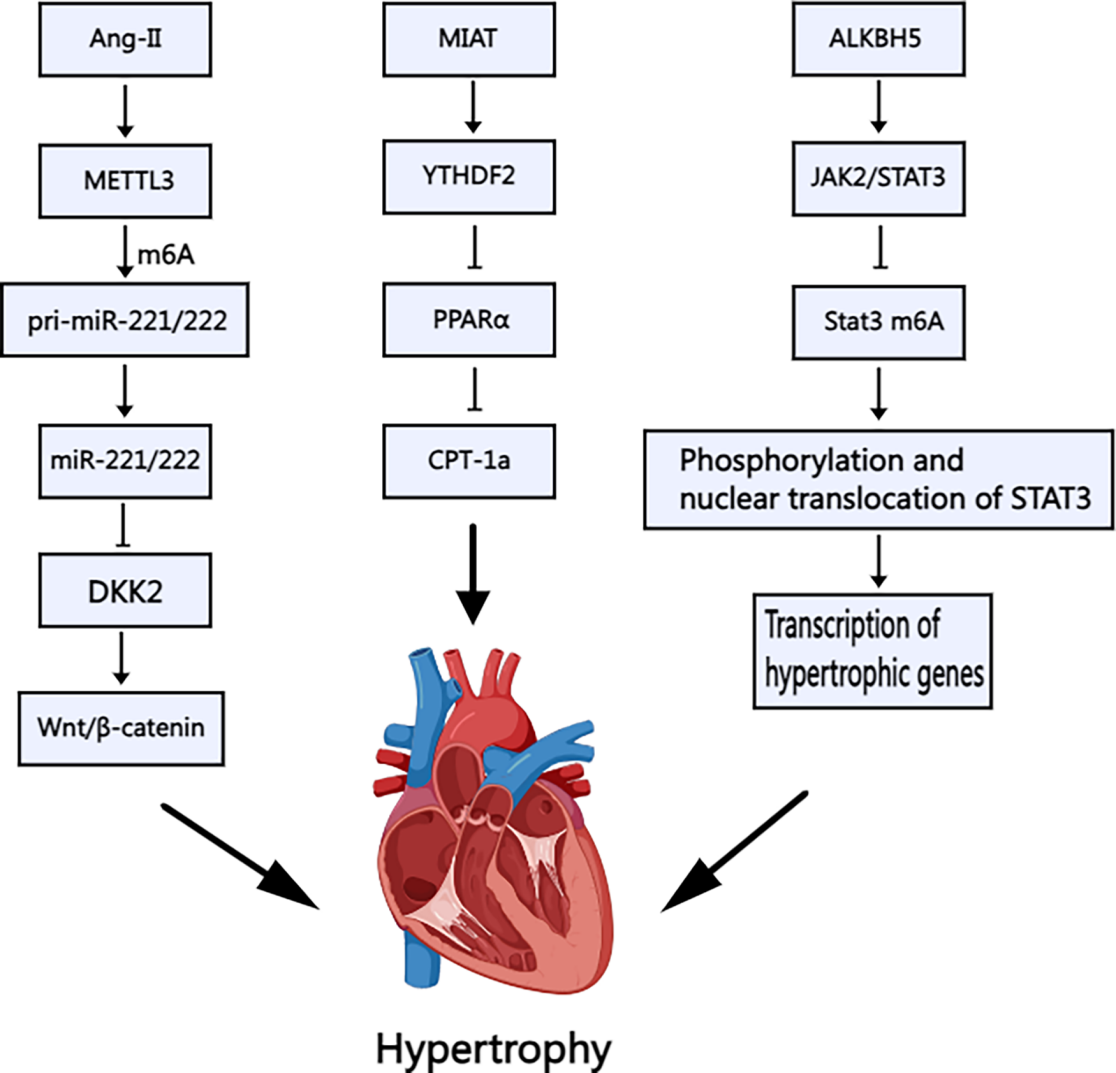
Schematic illustration of the mechanism of m6A methylation in cardiac hypertrophy. Ang-II: Angiotensin II; METTL3: Methyltransferase-like 3; DKK: Dickkopf, MIAT: Myocardial Infarction–Associated Transcript; YTHDF2: YT521-B Homology Domain Family 2; PPARα: Peroxisome proliferator-activated receptorα; CPT-1a: Carnitine palmitoyl transferase-1a; ALKBH5: AlkB Homolog 5; JAK2/STAT3: Janus kinase 2-signal transducers and activators of transcription 3. Created with MedPeer (medpeer.cn).

Zhang et al. demonstrated that angiotensin II (Ang-II) positively regulates METTL3-mediated m6A modification, which in turn upregulates the methylation and expression of miR-221/222. This activation triggers the Wnt/β-catenin pathway, promoting cardiomyocyte hypertrophy (28) (Figure 2). Furthermore, Lu et al. found that USP12 enhances the p300 expression, leading to increased METTL3 expression. This signaling pathway was subsequently identified as being linked to Ang-II (29). A comprehensive analysis of METTL3-mediated m6A modifications in cardiac hypertrophy revealed that mRNAs encoding protein kinases (30), such as the mitogen-activated protein kinase family, exhibit m6A-specific enrichment. Conversely, suppressing METTL3-mediated m6A modification inhibits cardiac hypertrophy to some extent, and this phenomenon is correlated with ALKBH5. ALKBH5 plays a role in RNA demethylation, and its effects on cardiac hypertrophy are both beneficial and detrimental. An *in vitro* study by Chen et al. found that ALKBH5 could be elevated during the hypertrophic process in cardiomyocytes. Furthermore, ALKBH5 was found to activate the JAK2/STAT3 signaling pathway and mediate the demethylation of Stat3 (31) (Figure 2). The JAK2/STAT3 signaling pathway plays a pivotal role in the pathogenesis of various cardiovascular disorders. It regulates the differentiation of cardiac fibroblasts and facilitates the repair of myocardial infarction. It also mitigates the inflammatory profile associated with diabetic myocarditis (32–34).

Nevertheless, the relationship between the JAK2/STAT3 signaling pathway and dilated cardiomyopathy remains unexplored. However, STAT3 activation is believed to regulate inflammatory factors such as IL-6 and TNF-α (35), which are particularly important in the inflammatory progression of DCM. Aberrant JAK2/STAT3 signaling has been linked to enhanced fibroblast transformation, potentially leading to myocardial fibrosis and exacerbating cardiac dilatation and dysfunction. Moreover, STAT3 maintains mitochondrial integrity and resilience to oxidative stress, which is particularly relevant given the aberrant mitochondrial characteristics observed in patients with DCM. These mitochondrial abnormalities can disrupt energy supply and lead to overcompensation (36, 37). The JAK2/STAT3 signaling pathway can potentially mitigate cardiomyocyte injury and apoptosis by enhancing mitochondrial function. Therefore, research on this signaling pathway and its role in DCM can provide meaningful insights.

Furthermore, m6A may modify noncoding RNAs, influencing cardiac hypertrophy. Noncoding RNAs encompass a range of RNA molecules, including tRNAs, rRNAs, siRNAs, and long noncoding RNAs (lncRNAs). tRNA alterations have been linked to amino acid transport defects in primary dilated cardiomyopathy, with familial inheritance patterns. Yang et al. examined the levels of the lncRNA MIAT in cardiac hypertrophy and found that the overexpression of MIAT and the downstream response molecule Ythdf2 exacerbates cardiac hypertrophy (38) through the PPARα/CPT-1a signaling pathway (Figure 2). While microRNAs have been studied in this context, there is a paucity of targeted research on lncRNAs, warranting further attention. Due to the lack of a 5′m7G cap structure, circRNA was initially considered to be non-protein-coding RNA and therefore incapable of translation via a cap-dependent mechanism. However, studies have shown that circRNA can be translated through a cap-independent mechanism, including those involving m6A. The proteins encoded by circRNA may exhibit distinct biophysical properties compared to those encoded by mRNA. Once translated, these proteins can alter cell characteristics, potentially conferring new functions related to disease. Current research primarily focuses on the role of circRNA in cancer, while its involvement in cardiomyopathy remains to be explored (39).

### Other methylations in cardiac hypertrophy

In addition to the most prevalent m6A modification, researchers have investigated other methylations and their relationship with cardiac hypertrophy. m1A modifications are predominantly present in tRNAs. The enzyme TRMT6 and its isoforms, along with ribosomal RNA processing protein 8 (NLM), play crucial roles in the recognition and reading of m1A (40–42). YTHDC1, a member of the YTH protein family, is also involved in the recognition and binding of m1A modifications, regulating nuclear RNA splicing and transporter activity (43). Furthermore, the ALKBH family and FTO have been identified as key regulators of m1A modification (42) (Figure 1B).

M5C is ubiquitously distributed in mRNAs, tRNAs, rRNAs, and lncRNAs. The enzymes responsible for the “writing” of m5C are primarily members of the NSUN family. NSUN2 is the most significant m5C RNA methyltransferase, and its aberrant function has been implicated in numerous cancers and neurodegenerative diseases (44–46). NSUN3 and NSUN4 primarily regulate mitochondrial protein synthesis. Besides the NSUN family, DNA methyltransferase-like protein (DNMT2), a tRNA methyltransferase, also catalyzes m5C modification of cytosine in tRNA (Figure 1D). DNMT2 plays a critical role in maintaining tRNA stability and function. Yu et al. demonstrated that DNMT2 deficiency affects mutant phenotypes and maternal gene transmission. Furthermore, studies have linked DNMT2 to biological stress (47–49). The “erasure” of M5C is mediated primarily by ten-eleven translocation (TET) family proteins. TET proteins are well-characterized m5C demethylases that oxidize m5C in DNA to 5-hydroxymethylcytidine (5hmC) through the AM-AR and AM-PD pathways, with several oxygenation pathways serving as the underlying mechanism. Mutations in TET2 have been linked to the expansion of hematopoiesis in precancerous stages and alterations in the immune response in solid and hematological tumors. These mutations are heritable (50, 51). The Aly/REF export factor (ALYREF) facilitates the translocation of mRNA from the nucleus to the cytoplasm by recognizing and binding to m5C modifications. ALYREF plays a pivotal role in tumor development and remission as part of the HIF1-α signaling pathway. Wang et al. demonstrated that m5C modification of PKM2 mRNA may promote glucose metabolism in bladder cancer. Yang et al. identified ALYREF as a key factor involved in the modification of LINC02159, which advances lung cancer progression, as well as hepatocellular carcinoma (52–54). However, ALYREF's role in cardiovascular diseases remains largely unexplored.

M7G is a modification that is widely found in the 5′ end cap structure (5′ cap) of eukaryotic mRNAs (Figure 1C). RNA guanine-N7 methyltransferase (RNMT) is mainly responsible for catalyzing the methylation of the nitrogen atom at position 7 of guanine G, converting it into the M7G cap structure. Eukaryotic translation initiation factor 4E (elF4E) then binds to the m7G cap to facilitate the initiation of translation. Additionally, cap-binding proteins CBP80/CBP20 interact with the M7G cap. Research by Michael J. Osborne et al. revealed the formation of the m7G cap-eIF4E-RNMT trimeric complex. This was the first time that the two enzymes were found to bind directly to improve the homeostasis of modified RNA (55). The DCP1/DCP2 complex is the major decapitation enzyme that recognizes and removes the M7G cap, initiating mRNA degradation. This process is crucial for mRNA decay mechanisms, including nonsense-mediated mRNA degradation (NMD) and AU-rich region-mediated mRNA degradation (ARE-mediated decay) (56). Pseudouridine modifications are predominantly found in tRNAs, rRNAs, and snRNAs and enhance RNA stability and function. Nicola Guzzi et al. reported that Ψ-modified tRNA fragments inhibit aberrant protein synthesis and can serve as biomarkers for leukemia development (57). Additionally, dyskerin was shown to mediate rRNA and mRNA pseudo-uridylation in a H/ACA snoRNA-dependent manner (58, 59).

**Figure 3:**
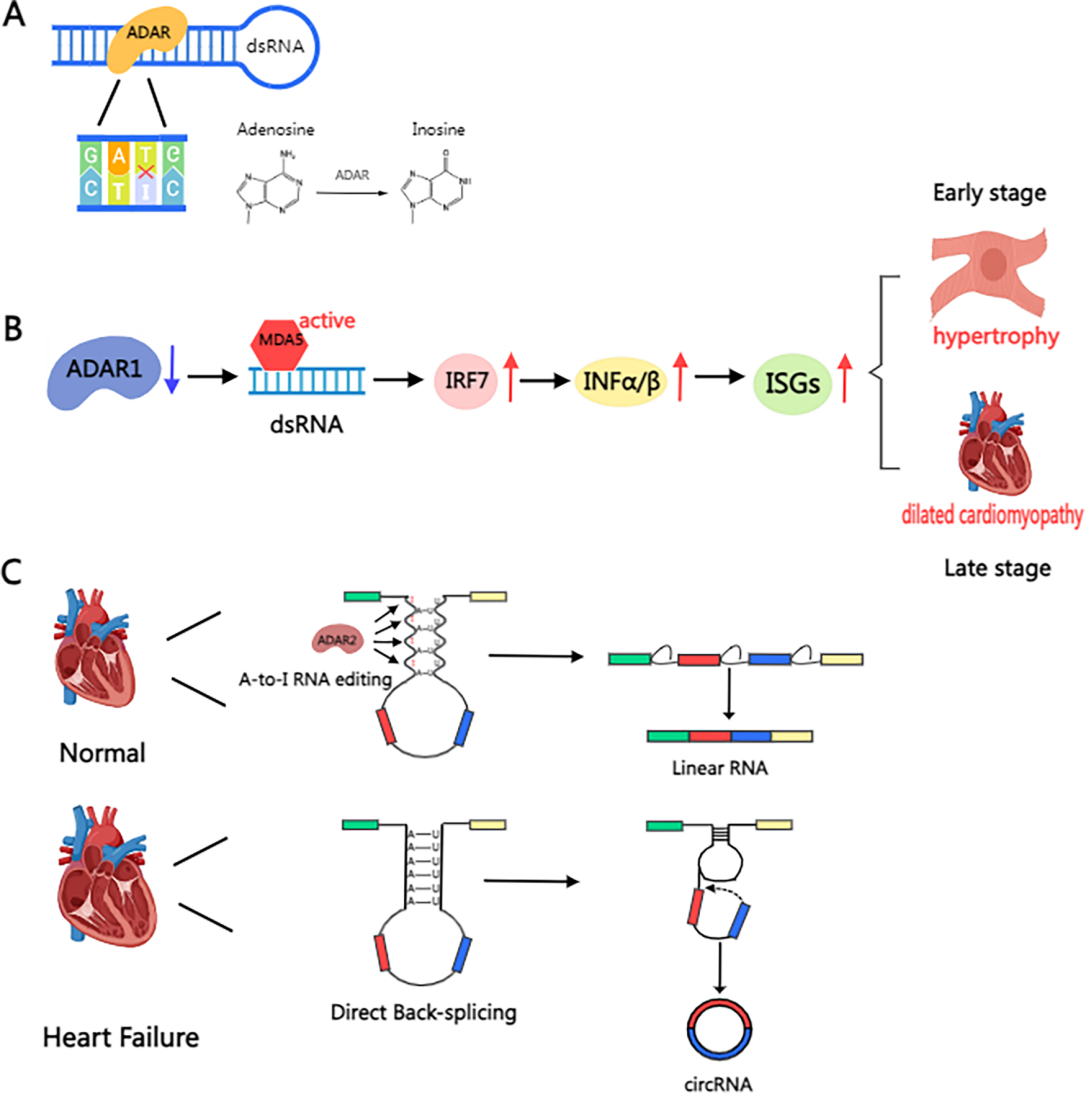
Schematic illustration of the mechanism of ADAR protein in the heart. **(A)** A-to-I RNA editing refers to the conversion of adenosine in RNA into inosine by adenosine deaminase (ADAR). **(B)** The deletion of ADAR1 prevents A-to-I editing in dsRNA, resulting in its recognition as “non-self” by MDA5, thereby activating IRF7, driving the secretion of type I interferon (IFN-α/β), inducing the expression of interferon-stimulated genes (ISGs), and triggering myocarditis and inflammatory cell infiltration. This induces cardiomyocyte hypertrophy in the early stage, eventually leading to dilated cardiomyopathy in the late stage. **(C)** Mechanisms of RNA editing and circular RNA formation in the failing human heart. In the healthy heart, ADAR2 maintains A-to-I editing in intronic Alu sequences, regulating typical pre-mRNA splicing leading to mRNA formation. In the failing heart, ADAR2 protein is reduced, resulting in reduced A-to-I editing in Alu elements. Alu repeats can pair and enhance circularization, leading to direct back-splicing and the formation of circular RNAs. Created with MedPeer (medpeer.cn).

Currently, there is a paucity of references to non-m6A methylations in the context of cardiac hypertrophy. However, adenosine-to-inosine (A-to-I) RNA editing is a prominent feature of cardiac disease, showing associations with all types of cardiac diseases. A-to-I RNA editing refers to the conversion of adenosine to inosine in RNA by adenosine deaminase (ADAR), which alters the molecular conformation of RNA, amino acid functioning and assembly, and the nature of the resulting protein (Figure 3A). ADAR1 also inhibits the ADAR1-dsRNA-MDA5 signaling pathway, which plays a crucial role in innate immune responses. This pathway is a potential target for alleviating cancer progression via suppression of ADAR1 overexpression (60). In cardiomyocytes, A-to-I editing of miR-34a eliminates mRNA repression. Tomer D. Mann and colleagues reported that ADAR1 enzymatic activity prevents IRF7-mediated autoinflammatory responses in the heart (61), alleviating the progression of early stages of cardiac hypertrophy and the corresponding symptoms of dilated cardiomyopathy (Figure 3B). Regulation of another enzyme, ADAR2, has been shown to affect the progression of cardiac hypertrophy. In the study by Kokot KE et al., upregulation of ADAR2 was found to alleviate circRNA level, which in turn regulated the progression of cardiac hypertrophy (Figure 3C). Furthermore, ADAR2 has been shown to impair smooth muscle contraction by impairing pre-FLNA mRNA editing (62, 63). A recent study showed that therapeutic inhibition of LincRNA-p21 may prevent cardiac hypertrophy (64).

## Various methylations in dilated cardiomyopathy

### Advances in the genetics and molecular mechanisms of DCM

Dilated cardiomyopathy is characterized by familial inheritance, toxin exposure, inflammatory transformation, and cardiac remodeling. Elucidating the underlying genetic and molecular mechanisms is crucial for understanding its pathogenesis. Genetic studies can help identify differences between normal and abnormal genes in DCM and explore the causes of wall thinning, chamber enlargement, and abnormal cardiomyocyte function.

The most common modes of inheritance for DCM are autosomal recessive and X-linked mitochondrial inheritance patterns (65), although other lower-probability mutations cannot be ruled out. Ramone Eldemire et al. identified the causative genes of hereditary DCM. The large number of causative genes are summarized as follows: genes related to mitochondrial metabolism (ATP-driven pumps), cellular junctions (anchoring proteins, laminin, pontins, integrins), and myocyte-associated genes (actin and myosin), and those related to RNA modification and transcription. There are also a small number of ion transport proteins and signaling pathway-related proteins. Mutations in the TTN (giant protein) gene in cardiac smooth muscle cells are the most common cause of inherited DCM. In addition, Ramone Eldemire reported that missense mutations in LMNA genes are an important cause of cardiomyocyte senescence, which in turn triggers DCM. Mutations in various RNA editing genes have emerged as key factors in DCM development, presenting a promising avenue for future research on methylations (66, 67).

RNA methylation can be used to examine the effect of abnormal RNA methylation on the inheritance of DCM. Effector molecules are involved in processes such as cell signaling, metabolism, and fibrosis. Studying the corresponding molecular mechanisms can provide insights into the pathological mechanisms of DCM. This in-depth understanding can help inform interventions altering the activity of effector molecules by enhancing or inhibiting RNA methylation to determine whether this can alleviate DCM. Here we will describe the role of RNA methylation in DCM and heart failure.

## Methylations in dilated cardiomyopathy

**Figure 4:**
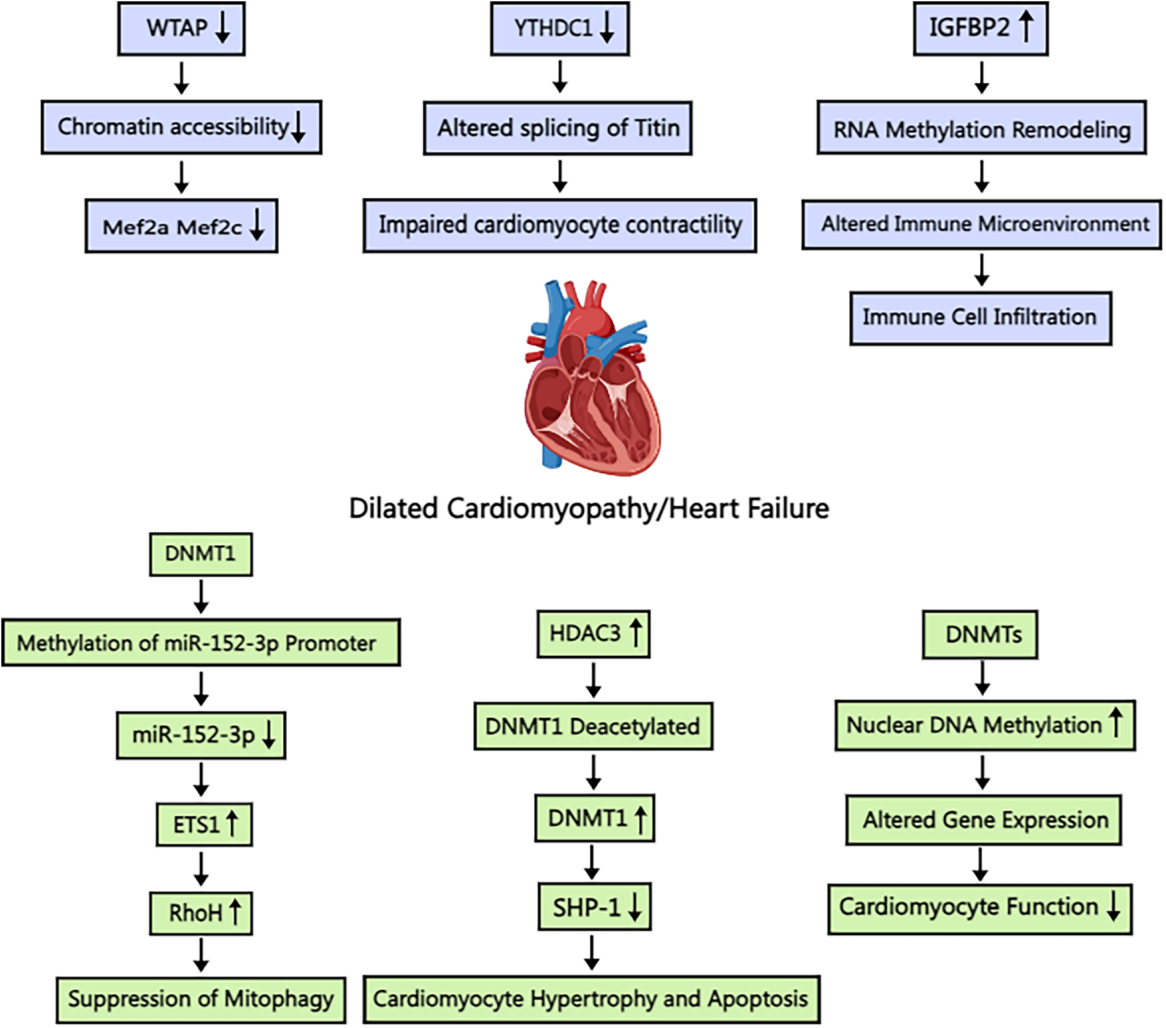
Schematic illustration of the mechanism of m6A and m5C methylation in dilated cardiomyopathy/heart failure. WTAP: Wilm's tumor 1-associating protein; Mef2a: myocyte enhancer factor-2a; Mef2c: myocyte enhancer factor-2c; YTHDC1: YT521-B homology-domain-containing protein 1; IGFBP2: Insulin-like growth factor binding protein 2; DNMT1: DNA methyltransferase 1; ETS1: E26 transformation specific-1; RhoH: Ras homolog gene family member H; HDAC3: histone deacetylase 3; SHP-1: Src homology domain 2-containing tyrosine phosphatase-1. Created with MedPeer (medpeer.cn).

Luo et al. explored the role of m6A RNA methylation in DCM's immune response (68) (Figure 4). Their study revealed the upregulation of four m6A regulators (METTL16, IGFBP2, TFEB, and HNRNPC) in DCM cardiomyocytes and downregulation of FTO (a demethylating enzyme) and WTAP (a coenzyme). Following immune system activation, lymphocytes and monocytes chemotactically accumulate in myocardial tissue, further increasing m6A expression. Luo et al. demonstrated a positive correlation between m6A expression and immune response. Notably, IGFBP2, which is highly expressed in cardiomyocytes and coincidentally corresponds to methylations, was found to affect signaling pathways, including but not limited to, the degradation of ROS as well as various intracellular metabolic pathways. Luo et al. further suggested that m6A-modified lymphocytes lead to cardiomyocyte fibrosis. However, the authors acknowledge that the underlying mechanisms are not fully understood and may involve additional inflammatory pathways. However, the left ventricular dysfunction caused by cytokine storm and monocyte infiltration is a plausible mechanism according to Luo et al. The role of m6A-modified B and T lymphocytes in DCM pathogenesis deserves further investigation. Kento Wada et al. demonstrated that reduced methylation of FKBP5 mRNA in leukocytes leads to increased FKBP5 transcription and translation, activating the classical regulator NF-κB and promoting IL-1β, which is elevated during cardiomyocyte necrosis (69). Elevated FKBP5 activity is also linked to chronic myocardial injury, influencing DCM progression. Studies in mouse models have underscored the critical role of m6A regulation in DCM. Shi L et al. showed that the absence of the m6A writer protein WTAP in cardiomyocytes is associated with the development of DCM (70) (Figure 4). Additionally, YTHDC1, a m6A reader protein, has been implicated in DCM pathogenesis, as cardiomyocyte-specific deletion of the Ythdc1 gene results in DCM in mice (71) (Figure 4).

While cellular-level research in DCM is prevalent, subcellular structural morphology studies are less common. Takashi Watanabe et al. employed electron microscopy to investigate cardiomyocyte nuclear structure in patients with DCM, revealing significant alterations (72) (Figure 4). In particular, they observed nuclear hypertrophy and extensive chromatin aggregation. Further investigation revealed increased m5C methylation in cardiomyocytes, predominantly localized to heterochromatin regions. This finding indicates that m5C methylation affects the transcription and translation of genes on chromatin. The authors concluded that m5C methylation affects myonode contraction, contributing to difficulties in heart muscle contraction. However, the localization of the gene remains unspecified. DNMT3a can also affect m5C methylation. Yu et al. reported that activated expression of DNMT3 impairs cardiomyocyte contraction (73), which ultimately leads to significant pathological changes associated with DCM or heart failure. Abnormal methylation of chromosomes, summarized above, causes heterochromatin aggregation, which exacerbates the symptoms of DCM; however, increased demethylase activity may not alleviate DCM symptoms. Tram Anh Tran et al. observed that inhibition of the Jumanji histone demethylase resulted in reduced histone demethylase function and increased histone methylation in cardiomyocytes (74), improving cardiac condition. Wang et al. also reported that JMJD3 inhibits SESN2 expression, leading to adriamycin-induced cardiomyopathy (75).

Future studies investigating chromosomal abnormalities and DCM progression should look at histone acetylation and methylation, as some of the DNA promoter methylation studies have reported conflicting results. Anupam Mittal et al. observed no significant difference in DNA promoter methylation between cardiomyocytes of DCM patients and normal humans (76). However, Sheng et al. suggested that DNA promoters as a whole were hypomethylated. Bong-Seok Jo also verified this notion but did not provide results from the control group (77). The authors believe that there are considerable inter-individual differences in the degree of DNA promoter methylation and that such epigenetic modifications are quite different from substantial gene mutations. Therefore, their studies may be quite misleading.

Julian L.P. Morivar et al. investigated the molecular mechanisms underlying myocardial fibrosis induced by fibroblasts, a key factor in DCM. Their study revealed that DNA methylation of LMNA and damage to the nuclear fibrillar layer trigger chromosomal remodeling in two ways. This epigenetic alteration contributes to the deregulation of a large number of genes, suggesting a combinatorial effect that drives DCM development (78). Sirisha M Cheedipudi et al. reported that m6A methylation in the lamina propria-associated structural domain (LAD) was significantly different from that in surrounding subcellular structures (79), with the most prominent methylation being that of TP53 (tumor protein 53), which affects the signal transduction pathway. Lucas Becker et al. explored the fibrotic characterization of the intercellular matrix of cardiomyocytes via Raman fiber spectroscopy, further defining the single-cell environment and improving the accuracy (80). It is envisaged that more advanced techniques will be used to detect methylation as science progresses. To halt the fibrotic process in cardiomyocytes, Wu et al. found that the combination of 3-methyladenine and the caspase 8 inhibitor zVAD-fmk alleviated inflammatory symptoms and prevented further aggravation of the fibrotic process (81). The transcription, mRNA modification, and stabilization of 3-methyladenine and caspase-8-related genes are associated with m6A methylation. However, Wu et al. did not elaborate on the detailed role of m6A in the response pathway.

Metabolic changes occur in all heart diseases, including cardiac hypertrophy and DCM. Chen et al. explored alterations in glycolysis during DCM progression and the molecular regulatory mechanisms involving methylation (82). They reported significantly increased expressions of the m6A methylation-related genes RBMX and ALKBH5 in DCM and that both RBMX and ALKBH5 were involved in signaling pathways such as the Toll-like receptor, complement receptor, and cytokine receptor. Upregulation of these two genes can be observed in cardiomyocytes from DCM patients. The enrichment analysis corroborated this conclusion. A comprehensive study of metabolic responses in DCM cardiomyocytes by Mark Epping et al. revealed the involvement of hypermethylated DNA promoters in oxidative metabolism, while promoter hypomethylation was found to stimulate glycolytic pathways (83). Revatitapu et al. reported aberrant upregulation of ATPase phospholipid transporter carrier methylation in damaged cardiomyocytes (84), demonstrating that methylation affects not only cardiac cell energy production in DCM but also energy transport and release.

Other studies have focused on the tricarboxylic acid cycle and amino acid and lipid pathways. Robert O Ryan found that impairment of the isoleucine pathway resulted in abnormally high levels of 2-ethylhydropropropenoic acid (2-EHA), a by-product of the blocked tricarboxylic acid cycle, in the myocardium (85). These findings provide potential biomarkers of metabolic abnormalities in DCM patients. Notably, the levels of other tricarboxylic acid reactants (products), such as α-ketoglutarate and succinate, which are significantly increased in DCM, can also be used as biomarkers. However, Young et al. reported no significant increase in pyruvate levels, which may be attributed to the fact that the glycolytic pathway itself is affected (86). Methylation of amino acids and proteins extends beyond metabolic pathways to structural proteins like troponins. Donatus O Onwuli et al. made a groundbreaking discovery, revealing that cardiac troponins in DCM undergo arginine methylation (87). This modification has profound results: rapid hypertrophy of cardiomyocytes and a greatly reduced inhibitory effect. These findings highlight arginine methylation of cardiac troponins as a potential biomarker for DCM. A recent study by Li et al. demonstrated the pivotal role of succinate dehydrogenase (SDH) in regulating the equilibrium between fatty acid metabolism and glycolysis in patients with heart failure (88). They found that succinate induces DNA hypermethylation, which in turn suppresses FAO gene expression in the myocardium. Notably, the FAO gene is a well-known demethylase, underscoring the importance of controlling SDH activity in heart failure management.

## Methylations in heart failure

Heart failure is the end stage of many heart diseases, and its main symptoms are dyspnea, edema of the abdomen and lower limbs, weakness and fatigue, and palpitations. Heart failure is categorized as systolic heart failure (HFrEF), diastolic heart failure (HFpEF), and intermediate heart failure (89, 90). Systolic heart failure is characterized by impairment of the contractile function of the heart, impairing the pump function. It is often observed in the advanced stages of DCM. Diastolic heart failure refers to the impairment of the heart's diastolic function, where the heart is unable to relax sufficiently to hold enough blood (91–93). It is often associated with hypertension and cardiac hypertrophy, and its main feature is a near-normal ejection fraction. Edema of the abdomen and lower limbs associated with heart failure causes swelling and fluid retention, i.e., inadequate blood supply. The severity of edema correlates with the severity of heart failure (94–96). Heart failure can also be classified as ischemic or nonischemic, corresponding to heart attack and chronic heart disease, respectively. This article focuses on the epigenetic aspects of DCM, excluding acute heart failure with predominant infarction. The authors acknowledge that chronic heart failure methylation deserves further discussion. Heart failure research has seen significant advancements in recent years, spanning from pathophysiology to molecular mechanisms. This article's selection prioritized novelty and recency, drawing from recent developments and reviews from the past year to ensure timeliness and validity.

In heart failure, cardiomyocytes are unable to produce sufficient energy due to impaired aerobic oxidation, necessitating compensation through alternative metabolic pathways. Sun et al. reported that L-NAME inhibition of nitric oxide synthase (NOS) impairs the conversion of L-arginine to L-citrulline, reducing nitric oxide production. NO promotes vasodilation and regulates myocardial contractility and metabolic status (97). Therefore, patients with hypertension and heart failure may exhibit defects in NO signaling. Cardiomyocytes in heart failure, similar to those in DCM, exhibit hypomethylation of glycolysis pathway promoters at the metabolic level. Liao et al. identified the locations of differentially methylated genes in the myocardia of patients with nonischemic heart failure. Notably, DNMT3a and DNMT3b do not play a role in heart failure. Furthermore, knocking down DNMT3a/3b upregulates gene promoters that are largely unmethylated, suggesting alternative epigenetic mechanisms (52, 98). Furthermore, the DNMT family of proteins influences the progression of heart failure through a multitude of pathways. Wang et al. demonstrated that histone deacetylase 3 (HDAC3) inhibits SHP-1 expression through the deacetylation of DNAmethyltransferase 1 (DNMT1), thereby promoting heart failure (99) (Figure 4). Wu et al. demonstrated the protective effect of myocardial-specific knockout of Dnmt1 against doxorubicin-induced heart failure. Dnmt1 deficiency limited gene expression reprogramming, activated pathways related to myocardial protection and anti-apoptosis, and significantly enhanced cardiomyocyte resistance to pathological stress. Additionally, Dnmt1 knockout altered cardiac methylation status, impacting gene expression. These findings further highlight the crucial role of methylation modification in the pathogenesis of heart failure (100).

Fibrosis occurs in senescent cardiomyocytes or cardiomyocytes exposed to inflammation. Cardiac fibrosis involves excessive accumulation of extracellular matrix (ECM) in cardiomyocytes. Wu et al. reported that YTHDF1 promotes AXL translation and thus cardiac fibrosis (101). Tatsuyuki Sato et al. suggested that hypoxia-induced cardiac fibrosis is associated with the expression of HIF-1α in human cardiac fibroblasts, leading to the activation of profibrotic genes (102). Furthermore, myocardial fibrosis is associated with transcriptional repression and increased Ras GTP activity due to RAS protein activator 1 (RASAL1) promoter methylation. These findings suggest that promoter methylation of the Ras protein family is associated with cardiac fibrosis. Li et al. reported that in addition to the downregulation of Rasal1 activity, cardiac fibrosis can also be alleviated by inhibiting RASSF1A activity (103). Additionally, Lauren Kerrigan et al. observed hypomethylation of the integrin β-like 1 (ITGBL1) gene in the heart (104). ITGBL1 is expressed predominantly in fibroblasts and plays a role in cardiac fibroblast migration, contributing to increased fibrosis. ITGBL1 is associated with collagen biosynthesis pathways in the ECM and profibrotic gene expression.

Qi et al. reported differences in the degree of DIO3-FA27 promoter methylation between patients with varying degrees of heart failure. They subsequently investigated the expression and function of DIO3-FA27 in the heart (105). Their findings indicated that the degree of promoter methylation was correlated with clotting time and fibrin degradation, suggesting its potential role as a biomarker of heart failure progression and severity. Zhang et al. also investigated the effect of DIO3 methylation patterns on heart failure progression (106). They observed lower DIO3 promoter methylation levels in patients with early-stage heart failure compared to that in normal subjects. Furthermore, methylation was found to gradually decrease with disease progression, affecting coagulation and ultimately disrupting the internal homeostasis of the heart. Liu et al. reported aberrant methylation of the glutathione peroxidase 3 (GPX3) promoter region in patients with heart failure (107). However, further research is required to elucidate the precise role of GPX3 promoter region methylation in patients with chronic heart failure. The authors posit that aberrant methylation of the GPX3 promoter impairs the efficacy of GPX3, leading to cardiomyocyte damage owing to incomplete removal of oxygen produced by cardiac hypertrophy, DCM, and myocarditis associated with heart failure.

Mitochondrial dysfunction, a common feature of heart failure, results in reduced energy production, blocked transport pathways, increased necrosis, and ultimately cardiomyocyte death. Given that DNMT3a is implicated in the methylation of factors involved in glycolysis and aerobic oxidation, an increase in DNMT3a activity may lead to mitochondrial abnormalities. Deng et al. reported that DNMT1 can modify microRNA-152-3p and influence its expression (Figure 4). This, in turn, affects the expression of Ras homologous gene family member H (RhoH), reduces cardiomyocyte viability, and promotes mitochondrial autophagy in cardiomyocytes. These findings indicate that the DNMT family of proteins may be involved in the overall metabolism of cardiomyocytes, affecting both the expression of enzymes and factors associated with metabolic pathways as well as energy supply and utilization (108). Wang et al. reported that DNMT1-mediated modification of m5C attenuated mRNA transcript methylation of cardiomyocyte necroptosis-activating transcription factor 7 (Atf7) (109). This, in turn, led to the downregulation of the transcription of Chmp2A, an inhibitor of necroptosis, and ultimately contributed to necroptotic apoptosis in cardiomyocytes. Therefore, the HNEAP-DNMT1-ATF7-CHMP2A axis represents a potential therapeutic target for mitigating cardiac injury resulting from necrotic apoptosis in ischemic heart disease. Further research is warranted to fully elucidate its role in heart failure.

Furthermore, this may influence processes such as cellular damage, aging, and death (108). Zhang et al. reported increased mtDNA 6mA levels and METTL4 expression in heart failure patients. These changes can impair mtDNA transcription, ultimately leading to mitochondrial dysfunction (110). Notably, the transcription factor p53 was identified as a direct regulator of METTL4 transcription in response to HF. This study revealed a signaling pathway from METTL4 expression to mtDNA methylation and subsequent mitochondrial dysfunction.

Bioinformatics analyses have implicated m7G modification in cardiovascular diseases. Ma et al. investigated the expression and function of m7G RNA methylation regulators in heart failure, revealing a novel link between RNA m7G modification, immune microenvironment, and heart failure development. This study also screened related biomarkers (111).

Yu et al. investigated the role of METTL1 in heart failure, finding that METTL1 is upregulated in the hearts of patients with heart failure. Mettl1 knockout in mice was found to alleviate pressure overload-induced cardiac dysfunction, while heart-specific overexpression of Mettl1 led to cardiac remodeling. Mechanistically, METTL1 mediates the m7G modification of splicing factor SRSF9, increasing its expression and promoting alternative splicing and stability of the transcription factor NFATc4, thereby driving cardiac remodeling (112). However, further experimental and clinical studies are necessary to confirm the role of m7G modification and elucidate its underlying mechanism.

## Discussion

DCM is a complex cardiovascular disease characterized by left ventricular or biventricular dilatation and systolic dysfunction, ultimately progressing to heart failure. The etiology of DCM is multifactorial, with genetic factors playing an important role in its pathogenesis. Recently, methylation modification, a key epigenetic mechanism, has emerged as a focal point in the study of DCM and heart failure.

RNA methylation, especially m6A methylation, plays an important role in the pathological processes underlying DCM. m6A methylation plays a crucial role in regulating the expression, stability, and translational efficiency of key genes in cardiomyocytes. m6A modification influences the development of cardiomyocyte hypertrophy and fibrosis by modulating signaling pathways, notably the JAK2/STAT3 pathway. Aberrant activation of the JAK2/STAT3 pathway is associated with myocardial fibrosis and heart failure. STAT3 regulates inflammatory responses and maintains mitochondrial function and resistance to oxidative stress. Thus, m6A modification may represent a potential therapeutic target for controlling myocardial fibrosis and improving outcomes in DCM, given its impact on the JAK2/STAT3 pathway.

m6A methylation influences cardiomyocyte hypertrophy and fibrosis by regulating the expression of key genes and non-coding RNAs. For example, METTL3-mediated m6A modification promotes cardiomyocyte hypertrophy by up-regulating miR-221/222 expression, thereby activating the Wnt/β-catenin pathway. Additionally, ALKBH5 mediates the demethylation of STAT3 and regulates the expression of inflammatory factors (such as IL-6 and TNF-α) by activating the JAK2/STAT3 signaling pathway, thereby affecting the progression of cardiac hypertrophy. Furthermore, M6A methylation modulates cardiac hypertrophy by modifying non-coding RNA (such as lncRNA MIAT). For example, overexpression of MIAT exacerbates cardiac hypertrophy via the PPARα/CPT-1a signaling pathway.

In addition to m6A modifications, other types of RNA methylations have been implicated in the development of cardiomyopathy. m1A and m5C modifications have been shown to regulate RNA stability and translation. m5C modifications regulate myocardial fibrosis and metabolism, potentially affecting the expression of metabolism-related genes in cardiomyocytes and regulating myocyte energy metabolism, in turn affecting the development of DCM. Furthermore, the DNA methyltransferase (DNMT) family of proteins, particularly DNMT3A and DNMT3B, plays a pivotal role in heart failure and myocardial fibrosis by regulating gene expression through DNA methylation.

Metabolic dysregulation and mitochondrial dysfunction are hallmarks of heart failure pathology. m6A modifications significantly impact metabolic regulation in cardiomyocytes. For example, FTO demethylase expression is closely associated with metabolic alterations in patients with heart failure. FTO enhances the expression of fatty acid oxidation-related genes in cardiomyocytes, restores mitochondrial function, and optimizes energy metabolism by removing m6A modifications. Succinate dehydrogenase (SDH) plays a key role in regulating fatty acid metabolism and glycolysis homeostasis in DCM patients. Succinate induces DNA hypermethylation, thereby inhibiting the expression of FAO gene in the myocardium. These mechanisms offer a novel regulatory pathway for metabolic disorders in heart failure.

In the context of myocardial fibrosis, m6A modification has been shown to regulate the translation efficiency of fibrosis-related genes, influencing the binding of YTHDF family proteins. Research has demonstrated that m6A methylation affects cardiomyocyte metabolism, fibrosis, and apoptosis by regulating the expression of key genes and non-coding RNAs. For example, YTHDF1 promotes cardiac fibrosis by enhancing AXL translation. Additionally, DNMT1-mediated m5C modification leads to cardiomyocyte necroptosis by attenuating the mRNA transcriptional methylation of necroptosis-activated transcription factor 7 (Atf7). These studies indicate the pivotal role of RNA methylation in the occurrence and development of heart failure. With the advancement of m6A methylation sequencing technologies, such as miCLIP-seq, which enable accurate single-base methylation resolution, future studies may further elucidate the relationship between m6A methylation and cardiovascular disease.

## Conclusions

In conclusion, methylations play an integral role in the pathophysiology of DCM, cardiac hypertrophy, and heart failure. By regulating RNA stability, splicing, translocation, and translation, methylations affect cardiomyocyte structure and function. m6A methylation influences cardiomyocyte hypertrophy, fibrosis, apoptosis, and metabolism by regulating key genes and non-coding RNAs. Additionally, m1A and m5C methylation play important roles in cardiac hypertrophy and heart failure. Future research should elucidate the specific mechanisms of RNA methylation in DCM and heart failure, providing novel therapeutic targets and strategies. In summary, RNA methylation's role in DCM and heart failure is a complex, multi-level process involving multiple signaling pathways and molecular mechanisms. Further research is required to obtain a deeper understanding of the dynamics of methylation modifications in different cardiac stages and to develop therapeutic strategies that target these modifications to slow the progression of cardiomyopathy. In-depth study of the biological function and mechanism of RNA methylation can provide a better understanding of the pathological process of DCM and heart failure, and provide a theoretical basis for the development of new treatment methods.
